# Type 2 Diabetes Mellitus (T2DM) “Remission” in Non-bariatric Patients 65 Years and Older

**DOI:** 10.3389/fpubh.2019.00082

**Published:** 2019-04-12

**Authors:** Srikanth Tangelloju, Bert B. Little, Robert J. Esterhay, Guy Brock, A. Scott LaJoie

**Affiliations:** ^1^Department of Health Management and Systems Sciences, School of Public Health and Information Sciences, University of Louisville, Louisville, KY, United States; ^2^Ohio State University Wexner Medical Center, Columbus, OH, United States; ^3^Department of Health Promotion and Behavioral Sciences, School of Public Health and Information Sciences, University of Louisville, Louisville, KY, United States

**Keywords:** diabete mellitus, Type 2 diabete mellitus, remission, bariatric, medicare, 65 years and older

## Abstract

**Objective:** To analyze the factors associated with type 2 diabetes mellitus (T2DM) “remission” in non-bariatric Medicare patients 65 years and older.

**Research Design and Methods:** A retrospective cohort analysis of a Medicare Advantage health plan was conducted using administrative data. An individual was identified as T2DM if the individual had: ≥ 2 medical claims for T2DM coded 250.xx excluding type 1 diabetes; or ≥ 2 pharmacy claims related to T2DM; or ≥ 2 combined medical claims, pharmacy claims for T2DM in 12 months. A T2DM individual was in “remission” if they had no T2DM related claims for more than 12 months continuously. This is different from the standard American Diabetes Association (ADA) definition of remission which includes HbA1c values and hence is represented in quotation (as “remission”). 10,059 T2DM individuals were evaluated over a period of 8 years from 2008 to 2015. Cox proportional hazards was used to identify significant variables associated with T2DM “remission.”

**Results:** 4.97% of patients studied met the definition of T2DM “remission” in the study cohort. After adjusting for covariates this study found a number of variables associated with T2DM “remission” that were not previously reported: no statin use; low diabetes complications severity index score; no hypertension; no neuropathy; no retinopathy; race (non-white and non-African American); presence of other chronic ischemic heart disease (IHD) and females (*p* < 0.05).

**Conclusion:** T2DM “remission” in Medicare patients 65 years and older is observed in a community setting in a small proportion of non-bariatric patients.

Type 2 diabetes mellitus (T2DM) is generally considered as a chronic, incurable, progressive and life-long disease condition. T2DM disease management focuses primarily on glycemic control and management of the macrovascular and microvascular complications that may be associated with hyperglycemia (1, 2). However, a growing body of evidence indicates that T2DM remission can be achieved in certain individuals (3).

The American Diabetes Association (ADA) has defined the term remission as “…achieving glycaemia below the diabetic range in the absence of active pharmacologic or surgical therapy.” ADA classifies remission into one of the following three types: a) *partial remission*, defined as sub-diabetic hyperglycemia without active pharmacologic therapy or ongoing procedures for at least 1 year. Sub-diabetic hyperglycemia is characterized by HbA1_c_ < 6.5% and/or fasting glucose 100–125 mg/dL [5.6–6.9 mmol/l]; *b) complete remission*, defined as “normal” measures of glucose metabolism without active pharmacologic therapy or ongoing procedures for at least 1 year. Normal measure of glucose metabolism is when HbA1_c_ is in the normal range, < 5.7%, < 39 mmol/mol, fasting glucose < 100 mg/dL [5.6 mmol/l]); and *c) prolonged remission*, defined as complete remission for at least 5 years (4).

Current evidence on T2DM remission suggests that there are at least two key approaches for T2DM remission namely, metabolic/bariatric surgery and/or intensive lifestyle management (1). Of these two approaches, bariatric (“weight-loss”) surgery has been proved to be effective in treating T2DM patients whose BMI is 35 kg/m2 or higher (5) with a success of achieving T2DM remission between 24 and 95% (6, 7). Bariatric surgery is considered relatively safe with 30 day mortality between 0.1 and 1.1%. However, the risk of developing acute complications and long-term complications is high (17%). The rate of reoperation following bariatric surgery is around 7% and patients are often required to have a close postoperative medical monitoring (8–11).

Current understanding of T2DM remission among non-bariatric patients is very limited. A recent *post hoc* randomized controlled study from the Look AHEAD (Action for Health for Diabetes) and the 2014 Kaiser Permanente study have shown metabolic/bariatric surgery is not the only pathway to remission. Although rare, T2DM remission does occur in certain non-bariatric populations (3, 12, 13). However, very little is known about remission and factors associated with it.

Understanding factors predictive of remission within the Medicare patients 65 years and older is important because Medicare population differs from population at large by their age, life expectancy, and frequency of comorbidities (14), and have a significantly higher prevalence of T2DM compared to the population at large (15). Even though age itself is not a significant differentiator for bariatric outcomes, the higher occurrences of baseline comorbidities in the Medicare population might make this population less suitable for bariatric surgery (16, 17). Therefore, understanding these factors has substantial clinical relevance to the design of interventions which lead to remission. In addition, these data could be used to help design better T2DM management programs.

In order to identify remission as per the ADA definition, a researcher would need both pharmacy and laboratory data (HbA1_c_). However, laboratory test data are not always available to researchers, especially to those researchers who only rely on administrative claims data for their analytical work (18). Therefore, a modified definition for remission is offered in the present study. In this investigation, the modified definition of remission is enclosed in quotation marks (as possible “remission”).

In this study we estimate the rate of type 2 diabetes mellitus (T2DM) “remission” and analyze demographic, clinical and pharmacological factors associated with T2DM “remission” in non-bariatric Medicare patients 65 years and older.

## Research Design and Methods

### Study Design

This study was a retrospective cohort analysis using Medicare Advantage Prescription Drug (MAPD) health plan data. This administrative data includes patient level enrollment, medical claims and pharmacy claims data. These data were collected during the standard operations of a large multistate healthcare company. It was not originally collected for the purpose of research or any new study of human or animal subjects. Prior to the start of the study this study protocol was reviewed and approved by the University of Louisville Institutional Review Board. The study was also approved by the healthcare company's review board.

### Study Cohort

The federal government offers original Medicare insurance, which includes Medicare Part A (Hospital Insurance) and Medicare Part B (Medical Insurance). Part C (or Medicare advantage) are offered through private insurance companies. Part C includes the benefits of Part A, Part B and sometimes the optional Part D (prescription drug coverage) along with several choices of programs and services specific to each Managed Care Organization (MCO). Individuals can only be enrolled in Part C if they qualify for Medicare part A and part B. An individual may qualify for Medicare if they are at least 65 years old in the year of their Medicare enrollment and meet certain additional eligibility criteria. In addition, some individuals may qualify for Medicare for other reasons such as disabilities or end stage renal disease (ESRD). Individuals whose initial reason for Medicare enrollment was either disability or ESRD were excluded from the present study.

The large multistate healthcare company offers Medicare Advantage Prescription Drug Plan (MAPD) a Part C plan, which includes the benefits of Medicare Part A, Part B and Part D, to individuals. The healthcare company insured more than 2.4 million individuals who had MAPD coverage from January 1, 2008 to December 31, 2015. Of these ≥ 2.4 million individuals more than 1.7 million MAPD individuals had “age ≥ 65” as the original reason for Medicare enrollment. The remainder qualified under Medicare disability or ESRD eligibility provisions. Of these > 1.7 million patients there were 265,554 patients who had continuous MAPD coverage with the healthcare company from January 2008 to December 2015.

The initial sample after the above exclusions of 265,554 patients, additional exclusions were: (a) individuals who had a prior bariatric surgery (including gastric bypass, laparoscopic gastroenterostomy) (*n* = 619); (b) individuals with polycystic ovary syndrome (*n* = 131), and lipodystrophy (*n* = 718); (c) individuals with any diagnostic codes related to nephropathy were excluded because it indicates irreversible end organ damage. In the current study, the nephropathy exclusion resulted in excluding 41.3% of the study sample. Previous research suggested that the rate of chronic kidney disease (CKD) in individuals who are 65 years and older was 61%. CKD rate in the current study was likely lower because individuals with “disability” and “ESRD” as their initial reason for Medicare enrollment were excluded from the sample (19); (d) individuals who had pharmacy claims related to drug and/or steroid induced diabetes were excluded (*n* = 3,436); (e) individuals with type 1 diabetes were identified using ICD codes and excluded because type 1 diabetes has a different etiology from T2DM (*n* = 25,535); (f) individuals who had T2DM related diagnostics codes only during inpatient hospital stay and did not have T2DM diagnostic claims or T2DM related pharmacy claims in a non-hospital setting were excluded to account for potential surgery induced dysglycemia (*n* = 1,772); g) certain groups with contractual restrictions with the health plan prohibiting research (*n* = 2,238).

The definition of “remission” in this study was different from that used by ADA, because of the lack of HbA1_c_ data. An individual was encoded as being in “remission” if they met the following criteria: (a) had two or more T2DM related claims in previous years; (b) had no T2DM related claims in the given year; (c) upon “remission” T2DM individuals were required to continue to have no T2DM related claims until the end of study period, 2015. This requirement excluded individuals who experienced T2DM relapse. This study's focus was only on individuals who experienced continued “remission” and not intermittent “remission.”

**Figure 1:**
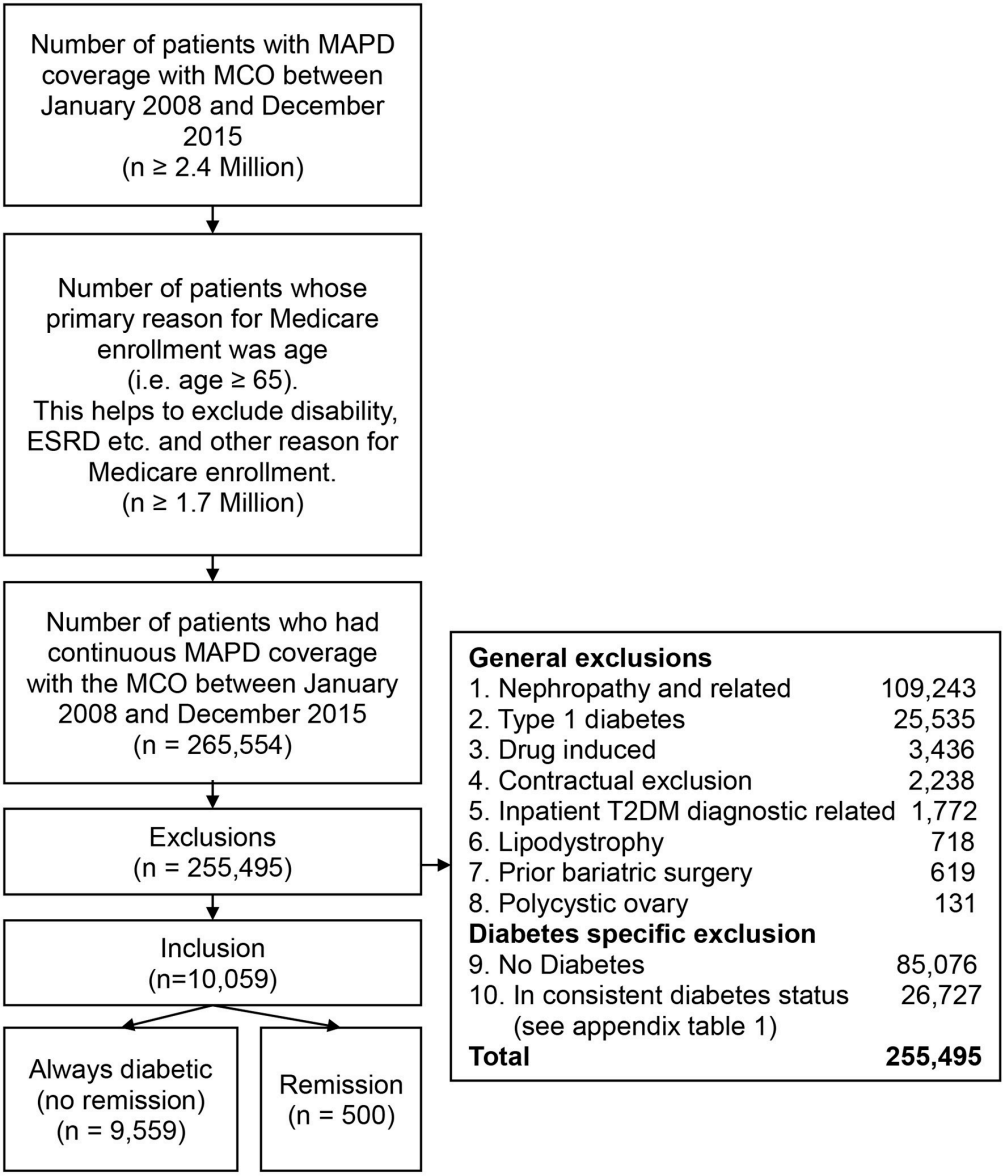
Inclusion and exclusion criteria.

Individuals were required to have T2DM in the year 2008, 2009, 2010, and 2011 and either continue to have diabetes (no “remission” group) or achieve “remission” (the “remission” group) to be included in the T2DM “remission” study cohort. T2DM relapse can occur in some individuals after “remission.” Individuals who had relapse after first “remission” were excluded from the study. The study cohort included a total of 10,059 patients. The “remission” group included 500 individuals and 9,559 patients in the no “remission group (Figure 1, Table A1 in Supplementary Material).

### Case Definitions

#### T2DM

T2DM was identified using administrative medical and pharmacy claims with the following criteria: at least two diagnoses of T2DM (International Classification of Diseases, Ninth Revision, Clinical Modification [ICD-9-CM] and/or (International Classification of Diseases, Tenth Revision, Clinical Modification [ICD-10-CM]; at least two prescriptions related to T2DM; at least 2 claims (medical or pharmacy) related to T2DM. A minimum of two claims was used in the identification of T2DM to exclude patients who were miscoded and patients who were suspected to have T2DM but were never formally diagnosed.

#### DCSI

The Diabetes Complications Severity Index (DCSI) models the severity of diabetes complication at a specific point in the patient's life. DCSI is calculated as previously described (18, 20). The final output is a patient's score (or index value) with a range from 0 to 13. An individual with DCSI score of 0 indicates the patient has the least severe diabetes. This DCSI score can be used to group the patients into high, medium and low diabetic severity.

#### Retinopathy

This study used ICD-9 and ICD-10 diagnostic codes to identify diabetic ophthalmologic disease, background retinopathy, other retinopathy, retinal edema, cystoid macular edema/degeneration (CSME), other retinal disorders, proliferative retinopathy, retinal detachment, blindness, vitreous hemorrhage). An individual with any of the above clinical conditions was encoded as having retinopathy.

#### Neuropathy

This study used ICD-9 and ICD-10 diagnostic codes to identify diabetic neuropathy, amyotrophy, cranial nerve palsy, mononeuropathy, Charcot's arthropathy, polyneuropathy, neurogenic bladder, autonomic neuropathy, gastroparesis/diarrhea, orthostatic hypotension. An individual with any of the above clinical conditions was encoded as having neuropathy.

#### Peripheral Vascular Disease (PVD)

This study used ICD-9 and ICD-10 diagnostic codes to identify diabetic PVD, other aneurysm lower extremities, PVD, foot wound and complication, claudication, intermittent, embolism/thrombosis lower extremities, gangrene, gas gangrene, ulcer of lower limbs. An individual with any of these clinical conditions was encoded as having PVD.

### Covariates

T2DM related clinical conditions were defined using administrative claims data as described previously using both ICD-9 and ICD-10 codes, where applicable (20). Demographic information (gender and race) was based on enrollment data. T2DM related pharmacy and statins use was determined using administrative pharmacy claims data.

### Statistical Analysis

Enrollment, medical and pharmacy claims data from 2008 to 2015 of more than 2.4 million patients were included in the data aggregation. The data aggregation was done in Hadoop (using Spark and Hive). Hadoop is an open source software framework for distributed processing and distributed storage of very large datasets (21). Processing was done using Apache Spark v1.6 because of the volume of the data included. The analytical dataset was created in a *counting process* input style (22, 23). Means and frequencies to generate patient profiles were calculated using Apache Spark v1.6. This data was then moved into SAS 9.2. (SAS Institute Inc., Cary NC) to calculate rate for T2DM “remission.” Cox proportional hazard models were specified to identify the significant variables associated with T2DM “remission” (22). The adjusted model included demographic variables (gender, and race), statin use, comorbidities (hypertension, hyperlipidemia, retinopathy, neuropathy, other chronic ischemic heart disease, other atherosclerotic cardiovascular disease and peripheral vascular disease) and DCSI score. The level of significance for all statistical tests was *p* < 0.05.

## Results

There were 10,059 people 65 years of age and older who had T2DM in 2008, 2009, 2010 and 2011. In the study period, there were 4.97% (95% CI, 3.05–8.00%) patients 65 years and older (*n* = 500) who met the definition of T2DM “remission” in the study cohort. At 2011, the average age of the “remission” group was 73.7 years, while that of the non- “remission” group was 72.6 years. In the “remission” group 62.2% were female, compared to 57.2% in the non-“remission” group (Table 1).

**Table 1:** Baseline demographic and T2DM “remission” characteristics.

**Characteristic**	No “Remission”* (*n* = 9,559)	**“Remission”^†^ (*n* = 500)**	***P*-value**
Age, mean (SD), years	72.6 (5.1)	73.7 (5.5)	< 0.0001
Female sex	57.2%	62.2%	0.0273
Race/ethnicity			0.3542
White	7,679 (80.4)	397 (79.6)	
Black	1,378 (14.4)	68 (13.6)	
Others	497 (5.2)	34 (6.8)	

Data are represented as n (%), unless otherwise mentioned. Year = 2008 ^*^Missing data in no “remission” cohort: Race (n = 5) †Missing data in “remission” cohort: Race (n = 1).

At year 2011, the T2DM “remission” group had significantly lower disease prevalence of hypertension, hyperlipidemia, retinopathy (overall, diabetic ophthalmologic disease, background retinopathy, and blindness), neuropathy (overall, diabetic neuropathy, polyneuropathy) and diabetic peripheral vascular disease. However, the “remission” group had higher cardiovascular disease (overall, myocardial infarction, aortic aneurysm and dissection) (*p* < 0.05) (Table 2).

**Table 2:** Baseline comorbidities.

**Characteristic**	No “Remission"* (*n* = 9,559)	**“Remission” (*n* = 500)**	***P*-value**
Hypertension	8,285 (86.7)	411 (82.2)	0.0040
Hyperlipidemia	8,125 (85.0)	402 (80.4)	0.0050
Retinopathy	1,187 (12.4)	32 (6.4)	< 0.0001
Diabetic ophthalmologic disease	830 (8.7)	17 (3.4)	< 0.0001
Background retinopathy	392 (4.1)	< 10	0.0010
Other retinopathy	138 (1.4)	< 10	0.6550
Retinal edema	67 (0.7)	< 10	0.0600
Cystoid macular edema/degeneration	64 (0.7)	< 10	0.2010
Other retinal disorders	40 (0.4)	< 10	0.9500
Proliferative retinopathy	61 (0.6)	< 10	0.0730
Retinal detachment	72 (0.8)	< 10	0.1550
Blindness	55 (0.6)	< 10	0.0220
Vitreous hemorrhage	21 (0.2)	< 10	0.4110
Neuropathy	1,801 (18.8)	68 (13.6)	0.0030
Diabetic neuropathy	1,395 (14.6)	46 (9.2)	0.0010
Mononeuropathy	356 (3.7)	19 (3.8)	0.9310
Charcot's arthropathy	< 10	< 10	0.1870
Polyneuropathy	820 (8.6)	20 (4.0)	< 0.0001
Neurogenic bladder	15 (0.2)	< 10	0.8140
Autonomic neuropathy	78 (0.8)	< 10	0.3070
Gastroparesis/diarrhea	44 (0.5)	< 10	0.3950
Orthostatic hypotension	145 (1.5)	10 (2.0)	0.3930
Cerebrovascular	841 (8.8)	42 (8.4)	0.7590
Transient ischemic attack (TIA)	168 (1.8)	11 (2.2)	0.4660
Stroke	759 (7.9)	38 (7.6)	0.7840
Cardiovascular	2,925 (30.6)	174 (34.8)	0.0470
Atherosclerosis	572 (6.0)	36 (7.2)	0.2660
Other ischemic heart disease	112 (1.2)	< 10	0.3900
Angina pectoris	309 (3.2)	16 (3.2)	0.9680
Other chronic ischemic heart disease	1,846 (19.3)	94 (18.8)	0.7770
Myocardial infarction	81 (0.8)	10 (2.0)	0.0080
Ventricular fibrillation, arrest	732 (7.7)	43 (8.6)	0.4410
Atrial fibrillation, arrest	11 (0.1)	< 10	0.5920
Other atherosclerotic cardiovascular disease	138 (1.4)	10 (2.0)	0.3140
Old myocardial infarction	462 (4.8)	25 (5.0)	0.8650
Heart failure	503 (5.3)	34 (6.8)	0.1360
Atherosclerosis, severe	< 10	< 10	0.6090
Aortic aneurysm/dissection	121 (1.3)	17 (3.4)	< 0.0001
Peripheral vascular disease	1,074 (11.2)	60 (12.0)	0.5980
Diabetic PVD	524 (5.5)	16 (3.2)	0.0270
Other aneurysm, lower extremity	< 10	< 10	0.0090
Peripheral vascular disease	807 (8.4)	51 (10.2)	0.1700
Foot wound + complication	< 10	< 10	0.7460
Claudication, intermittent	623 (6.5)	46 (9.2)	0.0190
Embolism/thrombosis (lower extremity)	< 10	< 10	0.0440
Gangrene	< 10	< 10	0.6470
Gas gangrene	< 10	< 10	0.8190
Ulcer of lower limbs	98 (1.0)	< 10	0.3530
Metabolic	46 (0.5)	< 10	0.3230
Ketoacidosis	16 (0.2)	< 10	0.8630
Hyperosmolar	22 (0.2)	< 10	0.4480
Other coma	< 10	< 10	0.3960

*Data are expressed as n (%). Baseline year = 2011*.

*If the number of individuals in a cell were less than 10, the number is replaced with “ < 10”*.

In multivariate analyses, T2DM “remission” was associated with no statin use, low diabetes complications severity index score, no hypertension, no hyperlipidemia, no neuropathy, no retinopathy, race (Asian/Hispanic), presence of other chronic ischemic heart disease (IHD) and female gender (*p* < 0.05).

**Figure 2:**
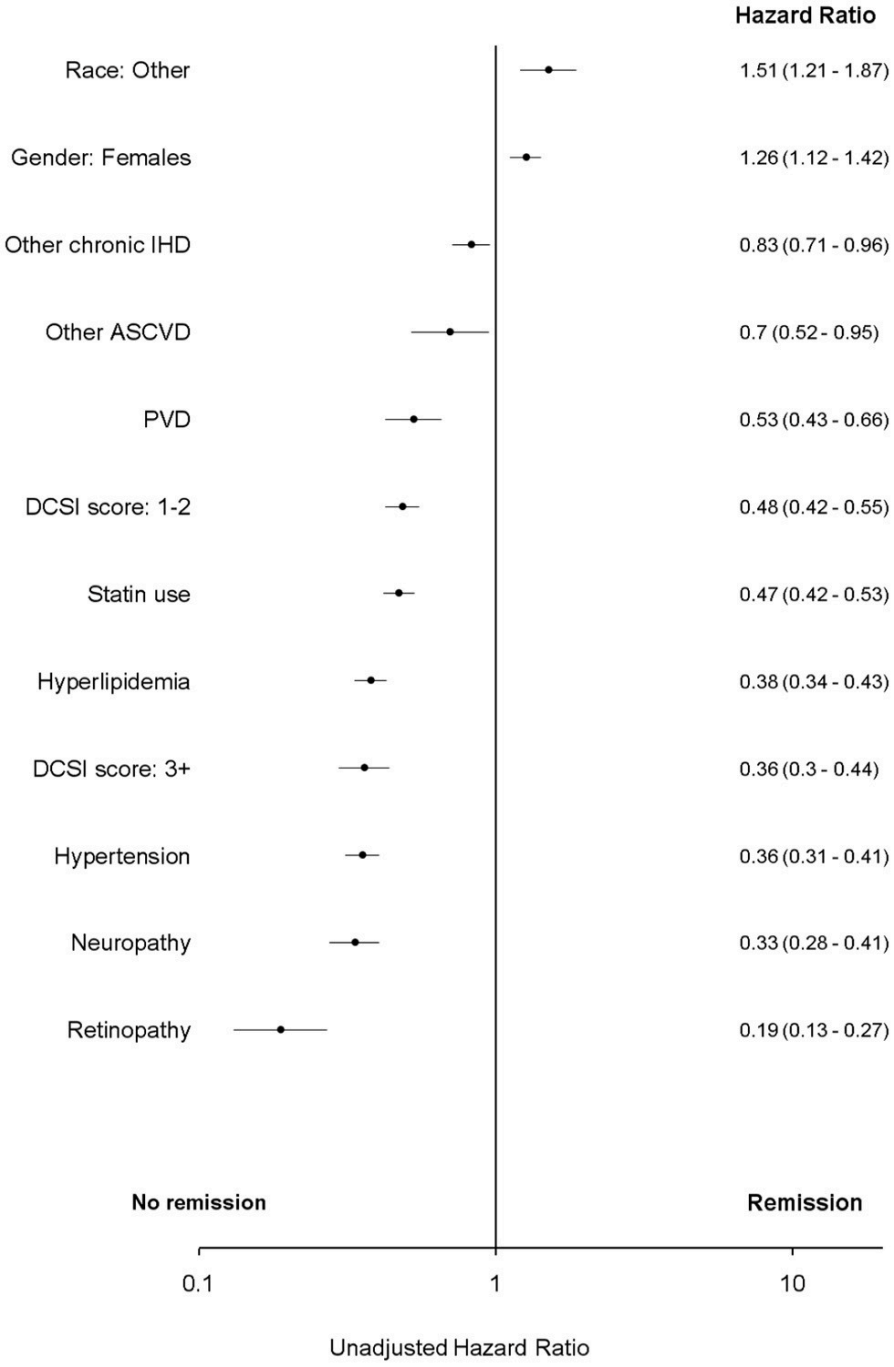
Unadjusted Cox proportional hazard for T2DM “remission.” ASCVD, atherosclerotic cardiovascular disease; DCSI, Diabetes complications severity index; IHD, ischemic heart disease. Race, other, non-white and non-African American; PVD, peripheral vascular disease.

**Figure 3:**
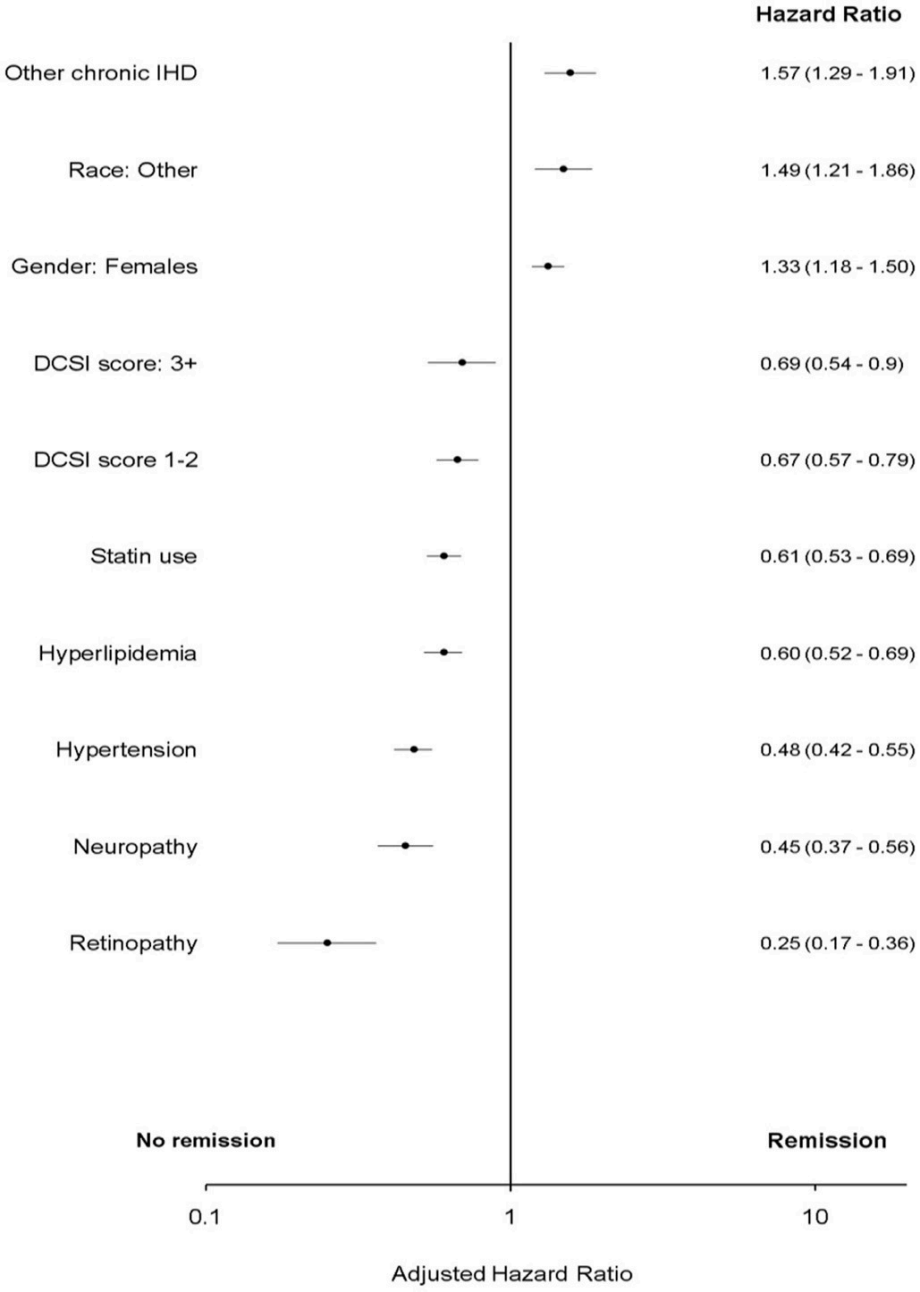
Adjusted Cox proportional hazard for T2DM “remission.” DCSI, Diabetes complications severity index; IHD, ischemic heart disease; Race, other, non-white and non-African American.

In bivariate analysis “remission” was associated with atherosclerotic cardiovascular disease and peripheral vascular disease (PVD). However, these associations were attenuated in multivariate analyses and were not significant (*p* > 0.05) (Figures 2, 3 and Table A2 in Supplementary Material).

## Conclusions

In non-bariatric patients 65 years and older with Medicare insurance, T2DM “remission” does occur. 4.97% (95% CI, 3.05% - 8.00%) of patients 65 years and older met the definition of T2DM “remission” in the study cohort. This was not significantly different from the 2.2% (1.34–3.54%) of the 2014 Kaiser Permanente report on ≥65 year old population (13). It is significantly different from the Look AHEAD study which reported T2DM remission incidence of 11.5% (95% CI, 10.1–12.8%) during the first year and 7.3% (95% CI, 6.2–8.4%) at 4 years in the intensive lifestyle intervention group. The percent “remission” in the present study did not differ from the 2.0% remission reported for the diabetes support and education control condition (DSE) group (*P* < 0.001) (12). The study presented here found that T2DM “remission” indeed occurs in Medicare patients 65 years and older who did not undergo bariatric surgery. These findings are consistent with previous findings and challenge the widespread notion that T2DM is irreversible in non-bariatric settings (13, 24, 25).

Consistent with the 2014 Kaiser and the Look AHEAD studies, this study also found that the absence of hyperlipidemia was associated with T2DM “remission.” As suggested in previous findings, hyperlipidemia, obesity, and impaired glucose tolerance may have a common biological pathway commonly called metabolic syndrome. These findings provide additional evidence of the role of managing impaired lipid metabolism to slow or reverse T2DM progression.

Further, this study evaluated multiple comorbidities related to macrovascular and microvascular comorbidities that are typically associated with T2DM. After adjusting for covariates this study found a number of variables associated with T2DM “remission” that were not previously reported: no statin use; low diabetes complications severity index score; no hypertension; no neuropathy; no retinopathy; race (non-white and non-African American); presence of other chronic ischemic heart disease (IHD) and females (*p* < 0.05).

### Gender

Globally, males are more likely to have T2DM than females (26, 27). This trend has changed over the last 50 years. Previously, females were more likely to have diagnosed T2DM, but in recent years, the increasing sedentary lifestyle of males is attributed to their increased prevalence of T2DM (28, 29). In line with these studies the current study found that females were more likely to have a T2DM “remission” compared to males.

### Race

Consistent with previous studies the current study found that Hispanics and other non-white race (excluding African American) were more like to have a T2DM “remission” in the Medicare 65 years and older population (*p* < 0.05) (30). This finding emphasizes the need for public health policies to develop culturally sensitive interventions to help ameliorate racial disparities of T2DM disease management especially in the elderly population.

### Hypertension

Previous studies indicated that hypertension and T2DM generally occur together and share several common risk factors such as obesity, inflammation, and insulin resistance. Hypertension and T2DM also share common pathways such as those associated with the sympathetic nervous system (SNS) and renin-angiotensin-aldosterone system (RAAS) (31). The present study found that even in Medicare patients 65 years and older population the absence of hypertension is associated with T2DM “remission” (*p* < 0.05).

### Statins

This study evaluated the role of statins on T2DM “remission” and found that the absence of statin use is associated with T2DM “remission” (*p* < 0.05). There is no previous literature on the role of statin in T2DM “remission.” However, its role on T2DM onset is well-established. Statin therapy is associated with a 9% increased risk of T2DM (32). Prior investigation also established that the level of statin potency is associated with T2DM onset (33).

### DCSI Score

The complications associated with T2DM usually develop simultaneously or consecutively rather than independently. A simple indicator for T2DM related complications may not have been a sufficient measure of T2DM disease progression. Therefore, in the present study, we used an indicator (or score), as previously described, to quantify the severity of diabetic complications (20, 34). This indicator is called the DCSI, and models the severity of diabetes complication at any given point of time in the patient's life. Using the DCSI score, we further analyzed the association between severity of T2DM and T2DM “remission,” and found that lower DCSI scores (i.e., individuals who have less severe diabetes) are associated with T2DM “remission.” This finding is important because DCSI can thus help in stratifying individuals with T2DM into groups based on score severity. This information can later be used for selecting individuals into disease management programs.

### Retinopathy

Retinopathy is one of the most common microvascular damage sites associated with T2DM. Diabetic retinopathy is a major public health problem, with approximately one third of T2DM patients developing some form of retinopathy (35). The healthy function of retina depends on availability of high amounts of oxygen, which in turn depends on intact blood-retinal barrier. The dysglycaemia associated with T2DM causes damage to this barrier by damaging both the inner retinal barrier and/or causing microvascular occlusions. The severity of such retinopathy might range from background retinopathy to complete blindness depending on the level of damage (36). Previous studies have recommended individuals with T2DM to control their blood sugar, blood pressure, and body fat to control the risk of development of advanced retinopathy (35).

Evidence in the present study found that the absence of any T2DM retinopathy was associated with T2DM “remission (*p* < 0.05). Taken in combination with previous recommendations, this study suggests that providers must educate T2DM about the possibility of “remission” by lifestyle modifications a self-incentive for enabling modified behavior, and that they still have a chance to reverse the progression of T2DM.

### Neuropathy

Neuropathy and diabetes are generally associated together. More than 50% of T2DM individuals have neuropathy and more than 50% of individuals with neuropathy have T2DM (37). The study presented here found that the absence of neuropathy was associated with T2DM “remission” in Medicare patients 65 years and older.

Neuropathy can be associated with several causes, including metabolic injury (hyperglycemia), compressive injury, ischemic injuries, inflammatory and/or immune response, and other comprehensive etiologies (38). This study did not evaluate the specific reason associated with neuropathy due to the lack of access to physician notes. In spite of this limitation, this finding provides valuable insight into designing public health interventions; for example, individuals with T2DM and without neuropathy may be educated about this finding to incentivize them to make behavior and lifestyle changes.

### Other Chronic IHD

IHD is the loss of elasticity and thickening of the coronary arteries, leading to progressive arterial insufficiency. Individuals with “other chronic IHD” were more likely to go into T2DM “remission.” This association is not completely understood. One explanation of this association is that there may be a small proportion of individuals in the study sample where the provider takes them off their diabetic medication due to a variety of reasons, such as the caregiver is less aggressive in motivating patients to achieve lower HbA1_c_ levels. Another explanation for this association is that individuals who have certain clinical conditions, such as those related to cardiovascular diseases, might be on constant alert to make healthier choices, especially due to the constant reminders that they might be receiving from their provider, insurance agency. These factors, and others, may be the reason why these individuals have a different outcome compared to those without this clinical condition.

### Limitations

There were several limitations to this study. First and most important, the study does not include clinical and laboratory findings such as physician notes and HbA1_c_ lab results, which would have provided a more accurate measure of T2DM “remission.” Therefore, a small proportion of individuals could have been asked by their doctors to stop taking T2DM medication for a variety of unknown reasons as noted. The probability of this occurring in the data is very low because even in those cases the physician will probably capture the T2DM related diagnostic code on the medical claims. Also, as with other similar retrospective studies there is a potential for error in information coded in the administrative data. As in other retrospective studies it is not possible to establish causation with such an analytic approach. The level or potency of the T2DM medications including statins was not evaluated in this analysis. DCSI is an unweighted composite index, and does not independently test individual outcomes associated with each complication associated with T2DM. Metabolic syndrome is not included in the study due to lack of data such as BMI and physician notes. T2DM onset age was not available to calculate T2DM disease duration. It is not known if an individual had bariatric surgery prior to 2008. Individuals in the study had T2DM from 2008 to 2011 and did not have bariatric surgery between 2008 and 2015. Typical remission rates are greatest by year 2 of bariatric surgery (39). Therefore, the likelihood of the observed “remission” being an outcome of a previous bariatric surgery in these patients is very low.

In conclusion, T2DM “remission” in patients 65 years and older with Medicare insurance is observed in a community setting in a small proportion of individuals who did not undergo bariatric surgery. This study helps establish additional evidence to the role of lifestyle intervention in T2DM progression and provides some insights into the possibility of using behavior and lifestyle interventions as alternate pathways for diabetes treatment and management.

Lifestyle interventions such as increased physical activity, low carbohydrate diet, cessation of tobacco smoking, and reducing alcohol intake are already recommended by providers to T2DM patients as a means to control glucose, blood pressure and cholesterol. These study results can further help providers discuss disease prognosis with their T2DM patients and in designing efficacious interventions to the right individuals at the right time. This will help design better T2DM management programs. For example, if a patient 65 years and older individual is diagnosed with T2DM and has not yet developed any chronic comorbidities such as neuropathy or retinopathy, but has modifiable comorbidities such as hypertension and hyperlipidemia, then their provider can use the insights provided in this study to advise their patients about their possibility to control their T2DM progression by participating in lifestyle intervention programs. DCSI score can be used as an index to further tailor these management programs.

These findings also have significant public health implications ranging from health policy, clinical guidance design, built environment and incentives to enable the success of these lifestyle intervention programs. Lastly, this study recommends further investigation into the lifestyle pathway for T2DM “remission” to improve our understanding of this beneficial alternative. Thus, it may be possible to increase the percent of individuals who might benefit from T2DM “remission.”

## Ethics Statement

The IRB for Human Subjects at the University of Louisville reviewed the study protocol and classified it as exempt status because previously collected information with no PHI.

## Author Contributions

ST, BL, RE, GB, and AL contributed to the analysis and interpretation of results. ST conducted all data manipulations as the study data was a proprietary secured database.

### Conflict of Interest Statement

The authors declare that the research was conducted in the absence of any commercial or financial relationships that could be construed as a potential conflict of interest.
