# Supplementary material for: Type 2 Diabetes Mellitus (T2DM) “Remission” in Non-bariatric Patients 65 Years and Older
Source: Front Public Health. 2019 Apr 12;7:82. doi: 10.3389/fpubh.2019.00082 (PMC6473045; doi:10.3389/fpubh.2019.00082)
Supplement: Supplementary file 1 [file Data_Sheet_1.docx]

Appendix: Table 1: T2DM “remission” definitions

| T2DM Diagnosis | | | | | | | |  |  |
| --- | --- | --- | --- | --- | --- | --- | --- | --- | --- |
| **2008**^§^ | **2009** | **2010** | **2011** | **2012** | **2013** | **2014** | **2015** | **PATTERN**^§§^ | **Description** |
| YES | YES | YES | YES | YES | YES | YES | YES | 11111111 | No “Remission”  (n = 9,559) |
| YES | YES | YES | YES | YES | YES | YES | NO | 11111110 | “Remission”  (n = 500) |
| YES | YES | YES | YES | YES | YES | NO | NO | 11111100 |  |
| YES | YES | YES | YES | YES | NO | NO | NO | 11111000 |  |
| YES | YES | YES | YES | NO | NO | NO | NO | 11110000 |  |
| YES | YES | YES | NO | NO | NO | NO | NO | 11100000 | “Remission”  (Excluded) |
| YES | YES | NO | NO | NO | NO | NO | NO | 11000000 |  |
| YES | NO | NO | NO | NO | NO | NO | NO | 10000000 |  |
| YES | YES | YES | YES | NO | NO | YES | YES | 11110011 | “Remission” + Relapse (Excluded) |
| YES | YES | YES | YES | NO | YES | NO | YES | 11110101 |  |

^§^NO = no type 2 diabetes diagnosis, and YES = type 2 diabetes diagnosis

**^§§^**0 = no T2DM and 1 = presence of T2DM

Appendix: Table 2 - Unadjusted and adjusted Cox proportional hazard for “remission”

|  | Any “Remission” (n = 500) | | | |  |
| --- | --- | --- | --- | --- | --- |
|  | Hazard Ratio (95% CI) | | | |  |
| Characteristic | n (%) | Unadjusted | P Value | Fully adjusted | P value |
| **Gender** |  |  |  |  |  |
| Males | 189 (4.4) | 1.0 (ref) |  | 1.0 (ref) | <0.0001 |
| Females | 311 (5.3) | 1.26 (1.12 - 1.42) | <0.0001 | 1.33 (1.18 - 1.50) |  |
| **Race** |  |  |  |  |  |
| White | 397 (4.92) | 1.0 (ref) |  | 1.0 (ref) |  |
| Black | 68 (4.7) | 0.93 (0.78 - 1.10) | 0.3936 | 1.01 (0.85 – 1.20 | 0.8849 |
| Other | 34 (6.4) | 1.51 (1.21 - 1.87) | 0.0002 | 1.49 (1.21 - 1.86) | 0.0002 |
| **DCSI Score** |  |  |  |  |  |
| Zero | 293 (7.7) | 1.0 (ref) | <0.0001 | 1.0 (ref) | <0.0001 |
| 1-2 | 152 (3.7) | 0.48 (0.42 - 0.55) |  | 0.67 (0.57 - 0.79) |  |
| 3+ | 55 (2.6) | 0.36 (0.29 - 0.44) |  | 0.69 (0.54 - 0.89) |  |
| **Statins** |  |  |  |  |  |
| No statins | 145 (9.32) | 1.0 (ref) |  | 1.0 (ref) | <0.0001 |
| Statins | 355 (4.17) | 0.47 (0.42 - 0.53) | <0.0001 | 0.61 (0.53 - 0.69) |  |
| **Clinical Conditions** |  |  |  |  |  |
| No hypertension | 33 (13.92) | 1.0 (ref) |  | 1.0 (ref) | <0.0001 |
| Hypertension | 467 (4.75) | 0.36 (0.31 - 0.41) | <0.0001 | 0.48 (0.42 - 0.55) |  |
| No hyperlipidemia | 25 (9.43) | 1.0 (ref) |  | 1.0 (ref) | <0.0001 |
| Hyperlipidemia | 475 (4.85) | 0.38 (0.34 - 0.43) | <0.0001 | 0.60 (0.52 - 0.69) |  |
| **Retinopathy** |  |  |  |  |  |
| No retinopathy | 411 (6.39) | 1.0 (ref) | <0.0001 | 1.0 (ref) | <0.0001 |
| Retinopathy | 89 (2.45) | 0.19 (0.13 - 0.27) |  | 0.25 (0.17 - 0.36) |  |
| **Neuropathy** |  |  |  |  |  |
| No neuropathy | 322 (6.85) | 1.0 (ref) | <0.0001 | 1.0 (ref) | <0.0001 |
| Neuropathy | 178 (3.32) | 0.33 (0.28 - 0.41) |  | 0.45 (0.37 - 0.56) |  |
| **Cardiovascular** |  |  |  |  |  |
| No other ASCVD | 476 (5.49) | 1.0 (ref) | 0.0202 | 1.0 (ref) | 0.3796 |
| Other ASCVD | 24 (1.74) | 0.70 (0.52 - 0.95) |  | 0.86 (0.62 - 1.19) |  |
| No other chronic IHD | 340 (5.45) | 1.0 (ref) | 0.0106 | 1.0 (ref) | <0.0001 |
| Other Chronic IHD | 160 (4.19) | 0.83 (0.71 - 0.96) |  | 1.57 (1.29 - 1.91) |  |
| **Peripheral vascular Disease** |  |  |  |  |  |
| No peripheral vascular disease | 380 (5.65) | 1.0 (ref) | <0.0001 | 1.0 (ref) | 0.1625 |
| Peripheral vascular disease | 120 (3.6) | 0.53 (0.43 - 0.66) |  | 0.85 (0.67-1.07) |  |

Fully adjusted model includes all variables in this table
*Missing data in No “remission” cohort: race (n = 5), in "remission" cohort: race (n = 1)
If the number of Individuals in a cell was < 10, the number is replaced with “< 10”

Data represented as n (%) for baseline year (2011)
